# Supplementary material for: The COVID-19 pandemic eroded system support but not social solidarity
Source: PLoS One. 2023 Aug 17;18(8):e0288644. doi: 10.1371/journal.pone.0288644 (PMC10434936; doi:10.1371/journal.pone.0288644)
Supplement: S1 File — (PDF) [file pone.0288644.s001.pdf]

Online Appendix for
The COVID-19 Pandemic Eroded System Support But Not Social Solidarity

Contents

A Question wordings **1**
A.1 Predictors – Covid Burden . . . 1
A.2 Predictors – Anomie . . . 1
A.3 Outcomes – Social solidarity . . . 2
A.4 Outcomes – System support . . . 3
A.5 Outcomes – Extreme discontent . . . 3
A.6 Outcomes – Additional measures . . . 4

B Descriptive Statistics **5**
B.1 Country statistics . . . 5
B.2 Demographics in the full and balanced panel samples . . . 6
B.3 Descriptive statistics for our outcomes . . . 8
B.4 Test-retest correlations and alpha reliability estimates . . . 10

C Model details and additional analyses **11**
C.1 Confidence intervals and regression tables for Fig 1 . . . 11
C.2 Regression tables accompanying Fig 3 . . . 15
C.3 Which demographic groups experience more burden and anomie during 2020? . . . 17
C.4 Estimating realistic changes in our predictors . . . 18
C.5 Descriptive Trends in Extreme Discontent . . . 18
C.6 Cross-country variation in 2FE models . . . 19
C.7 Additional outcome variables . . . 20
C.8 Splitting Covid Burden into component factors . . . 21
C.9 COVID-19 Burden, Anomie and Antigovernment Behavior Intentions – Replicating and extending Bartusevicius et al 2021. . . . 22
C.10 The benchmarked, single-item measure of anomie . . . 23

D Robustness checks **25**
D.1 Full CBS scale . . . 25
D.2 Testing the parallel trends assumption . . . 26
D.3 Controlling for Support for government . . . 27
D.4 Comparing one and two-way fixed effects models . . . 27
D.5 Replicating models without weights . . . 27

A Question wordings

Below we share the English language versions of our questions that were fielded in the USA. Naturally, where a question refers to the US or Americans, it is replaced with the country or nation of the respondent in all four countries.

A.1 Predictors – Covid Burden

Question: How much do you agree or disagree with the following statements?

1. {Health +} I am in good health despite the coronavirus crisis.
2. {Health –} I have felt extremely unwell as a consequence of the coronavirus crisis.
3. {Finance –}The coronavirus crisis has affected negatively my financial situation.
4. {Finance +} My finances are in good order despite the coronavirus crisis.
5. {Social life –} My social life has suffered a great deal due to the coronavirus crisis.
6. {Social life +} I have managed to stay in contact with my friends and family despite the physical distancing measures.
7. {Anomie –} The coronavirus crisis made me realize that all individuals can only rely on themselves.
8. {Anomie +} I believe the coronavirus crisis highlighted that the State can effectively protect its citizens.

The following two additional items have been omitted from all analyses unless otherwise noted, for being endogeneous with outcome variables.

1. {Democracy –} The extraordinary measures taken by the government in response to the coronavirus crisis make me concerned about my democratic rights.
2. {Democracy +} The government has not violated my democratic rights to fight the coronavirus beyond what is needed.

Scale: 1. Strongly agree; 2. Agree; 3. Somewhat agree; 4. Neither agree nor disagree; 5. Somewhat disagree; 6. Disagree; 7. Strongly disagree.

{ Info in brackets } indicates the five components of the index and whether the item is positively or negatively worded. Our main analyses exclude the Democracy component.

A.2 Predictors – Anomie

Question: How much do you agree or disagree with the following statements?

- My life is getting worse.
- These days I don't really know who I can count on.
- It is hard for me to know where I stand from one day to the next.
- Everything changes so quickly these days that I often have troubles deciding which are the right rules to follow.
- I often feel out of place.
- It is hard for me to decide what is the right thing to do.
- I feel all alone these days.
- I am sure I will find the right person who cares about me.
- I often feel discriminated against.
- My whole world feels like it is falling apart.
- I wish I were somebody important.
- I live a trapped life.
- I don't know what is expected of my life.

- I have no control over my destiny.
- My socioeconomic status determines my life.
- It is hard for me to understand what is going on in the world.
- No matter how hard I try in life, it does not make any differences.

Scale: 1. Strongly agree; 2. Agree; 3. Somewhat agree; 4. Neither agree nor disagree; 5. Somewhat disagree; 6. Disagree; 7. Strongly disagree.

A.3 Outcomes – Social solidarity

SUPPORT FOR REDISTRIBUTION

Question: Please read the following statements and indicate where you would place your views on this scale
1. Individuals should take more responsibility for providing for themselves.
... {answer options 2-9 which appear without a label are hidden for brevity}
10. The state should take more responsibility to ensure that everyone is provided for.

1. People who are unemployed should have to take any job available or lose their unemployment benefits.
...
10. People who are unemployed should have the right to refuse a job they do not want.

1. Incomes should be made more equal.
...
10. There should be greater incentives for individual effort.

SOCIAL TRUST

Question: Generally speaking, would you say that most people can be trusted or that you can't be too careful in dealing with people?

Scale: 1. Most people can be trusted; 2. Can't be too careful

TOLERATE IMMIGRANTS

Question: How much do you agree or disagree with this statement? "When jobs are scarce, employers should give priority to American people over immigrants"

Scale: 1. Agree strongly; 2. Agree; 3. Neither agree, nor disagree; 4. Disagree; 5. Disagree strongly

Question: Now we would like to know your opinion about the people from other countries who come to live in the US - the immigrants. How would you evaluate the impact of these people on the development of the US?

Scale: 1. Very good; 2. Quite good; 3. Neither good, nor bad; 4.Quite bad; 5.Very bad

Question: Please read the following statements and indicate where you would place your views on this scale
1. Immigrants take jobs away from Americans.
... {answer options 2-9 which appear without a label are hidden for brevity}
10. Immigrants do not take jobs away from Americans.

1. Immigrants make crime problems worse.
... {answer options 2-9 which appear without a label are hidden for brevity}
10. Immigrants do not make crime problems worse.

1. Immigrants are a strain on a country's welfare system.
... {answer options 2-9 which appear without a label are hidden for brevity}
10. Immigrants are not a strain on a country's welfare system.

1. It is better if immigrants maintain their distinct customs and traditions.
... {answer options 2-9 which appear without a label are hidden for brevity}
10. It is better if immigrants do not maintain their distinct customs and traditions.

REJECT SURVEILLANCE

Question: Please indicate if you think that the US government should or should not have the right to...

1. Keep people under video surveillance in public areas.
2. Monitor all e-mails and any other information exchanged on the Internet.
3. Collect information about anyone living in the US without their knowledge.

Scale: 1. Definitely should have the right; 2. Probably should have the right; 3. Probably should not have the right; 4. Definitely should not have the right

A.4 Outcomes – System support

SATISFACTION WITH THE POLITICAL SYSTEM

Question: On a scale from 1 to 10 where "1" is "not satisfied at all" and "10" is "completely satisfied", how satisfied are you with how the political system is functioning in your country these days? {options 2-9 unlabelled}

LEVEL OF DEMOCRACY

Question: And how democratically is this country being governed today? Again using a scale from 1 to 10, where 1 means that it is "not at all democratic" and 10 means that it is "completely democratic," what position would you choose? {options 2-9 unlabelled}

SUPPORT FOR DEMOCRACY

Question: In the following questions, various types of political systems will be described. For each one, would you say it is a very good, fairly good, fairly bad or very bad way of governing this country?

1. Having a strong leader who does not have to bother with parliament and elections.
2. {Having experts, not government, make decisions according to what they think is best for the country.}[7]
3. Having the army rule the country.
4. Having a democratic political system.

Scale: 1.Very good; 2.Fairly good; 3.Fairly bad; 4.Very bad
{The final support for democracy index flips the scores for items 1. and 3.}

PROUD CITIZEN

Question: How proud are you to be a US citizen?
Scale: 1.Very proud; 2.Quite proud; 3.Not very proud; 4.Not at all proud

A.5 Outcomes – Extreme discontent

NEED FOR CHAOS

Question: How much do you disagree or agree with the following statements?

1. I get a kick when natural disasters strike in foreign countries.
2. I fantasize about a natural disaster wiping out most of humanity such that a small group of people can start all over.
3. I think society should be burned to the ground.
4. When I think about our political and social institutions, I cannot help thinking "just let them all burn".
5. We cannot fix the problems in our social institutions, we need to tear them down and start over.

[7] As explained in the main text, we omit this answer option when forming the index.

6. I need chaos around me - it is too boring if nothing is going on.
7. Sometimes I just feel like destroying beautiful things.
8. There is no right and wrong in the world.

Scale: 1. Strongly agree; 2. Agree; 3. Somewhat agree; 4. Neither agree nor disagree; 5. Somewhat disagree; 6. Disagree; 7. Strongly disagree.

BELIEVING AND SHARING MISINFORMATION

Question: There are many stories about the coronavirus on the Internet. Some of these stories are true, others are not. Consider the following news story headline: "The coronavirus has been developed intentionally in a lab to be used as a bioweapon."

How much do you agree or disagree with the following two statements?

1. I think the story is true.
2. I might share the story on a social media platform (e.g. Facebook, Twitter).

Scale: 1. Strongly agree; 2. Agree; 3. Somewhat agree; 4. Neither agree nor disagree; 5. Somewhat disagree; 6. Disagree; 7. Strongly disagree.

POPULISM

Question: To what extent do you agree or disagree with the following statements?

1. Politicians should always listen closely to the problems of the people.
2. Politicians don't have to spend time among ordinary people to do a good job.
3. The government is pretty much run by a few big interests looking out for themselves.
4. Government officials use their power to try to improve people's lives
5. You can tell if a person is good or bad if you know their politics.
6. The people I disagree with politically are not evil.

Scale: 1. Strongly agree; 2. Agree; 3. Somewhat agree; 4. Neither agree nor disagree; 5. Somewhat disagree; 6. Disagree; 7. Strongly disagree.

A.6 Outcomes – Additional measures

AFFECTIVE POLARIZATION

Question 1: What do you feel when you think about people on the right in political matters?

Question 2: What do you feel when you think about people on the left in political matters?

1. Angry
2. Frustrated
3. Afraid
4. Hopeful
5. Enthusiastic
6. Proud

Scale: 1. Very strongly; 2. 3. 4. 5. 6. 7. Not at all.

{Affective polarization is calculated as the gap in emotions towards in-group and out-group (based on personal left-right placement). Scores to negative emotion items are flipped.

FINANCIAL PROSPECTS

Question: In your opinion, how hard is it to find good jobs in the community where you live?

Scale: 1. Good jobs are difficult to find. 2. Lots of some jobs, few of others. 3. There are plenty of good jobs 'available. 4. Don't know.

Question: Please think of your parents, when they were the same age as you. How is your standard of living compared to theirs? Is your standard of living. . .

Scale: 1. Much better; 2. Somewhat better; 3. About the same; 4. Somewhat worse; 5. Much worse; 6. Don't know;

Question: Please imagine your children when they reach the same age as you. How will their standard of living be compared to yours? Will their standard of living be

Scale:1. Much better; 2. Somewhat better; 3. About the same; 4. Somewhat worse; 5. Much worse; 6. Don't know;

GOVERNMENT SUPPORT

Question: How much do you agree or disagree with the following statements?

1. The government conducts a policy that harms the country's economy.
2. The government conducts a policy that is positive for the country.
3. The government has conducted the policies necessary to handle the coronavirus.
4. The government's response to the coronavirus has been too extreme.
5. Our country and our leaders have been united against the coronavirus.

Scale: 1. Strongly agree; 2. Agree; 3. Somewhat agree; 4. Neither agree nor disagree; 5. Somewhat disagree; 6. Disagree; 7. Strongly disagree.

B Descriptive Statistics

B.1 Country statistics

Table OA1 below reports macro-level statistics from the World Bank (GDP per capita PPP) and the V-Dem Institute (all others), to give a broad overview of the differences across countries. All V-Dem indicators are based on expert ratings.[8]

- V-Dem's **Liberal Democracy Index** scores countries on how close they get to the ideal of liberal democracy. The LDI "judges the quality of democracy by the limits placed on government", by incorporating the rule of law, civil liberties, freedom and fairness of elections etc.
- V-Dem's **Political Polarization** measures to what extent to which political differences affect social relationships beyond political discussions.
- **GDP per capita** on purchasing power parity from the World Bank.
- V-Dem's **Health Equality** measure asks "to what extent is high quality basic healthcare guaranteed to all, sufficient to enable them to exercise their basic political rights as adult citizens?"
- V-Dem's **Government effectiveness** combines into a single grouping responses on the quality of public service provision, the quality of the bureaucracy, the competence of civil servants, the independence of the civil service from political pressures, and the credibility of the government's commitment to policies. The main focus of this index is on "inputs" required for the government to be able to produce and implement good policies and deliver public goods.
- V-Dem's Pandemic Violations of Democratic Standards Index (**PanDem**) captures the extent to which state responses to Covid-19 violate democratic standards for emergency responses. Edgell, Amanda B., Jean Lachapelle, Anna Lührmann, ... and Staffan I. Lindberg. 2020. Pandemic Backsliding: Democracy During Covid-19 (PanDem), Version 6. Varieties of Democracy (V-Dem) Institute, www.v-dem.net/en/our-work/research-projects/pandemic-backsliding/.

[8]Coppedge, Michael, John Gerring, Carl Henrik Knutsen, Staffan I. Lindberg, ... and Daniel Ziblatt. 2021. "V-Dem Codebook v11.1" Varieties of Democracy (V-Dem) Project.

Table OA1: Country level statistics from Varieties of Democracy (V-Dem) and the World Bank

	Denmark	Hungary	Italy	USA
Liberal Democracy	0.88	0.37	0.79	0.73
Political polarization	0.04	0.95	0.79	0.93
GDP per capita	60,908	15,899	31,676	63,543
Health equality	0.96	0.60	0.93	0.47
Government effectiveness	0.99	0.62	0.62	0.87
PanDem score	0	0.3	0.15	0.2

B.2 Demographics in the full and balanced panel samples

Figure OA1 below reports the distribution of demographic variables (sex, education, age, and region) in all four countries for both full samples (with all data collected) and balanced samples (including only those who participated in all 3 waves), and for both unweighted and weighted samples. It is noteworthy that with the exception of the Hungarian data, the unweighted samples are reasonably close to population margins (the green and red marks are close to each other). It is also noteworthy that attrition in the balanced panel sample was not systematic by demographic attributes. The most recognizable pattern is that attrition slightly exacerbated existing skews in sex and age in Hungary, and age in the US. Finally, it is clear that the entropy weights we rely on in all analyses are very successful in bringing all demographics distributions to the population margins.

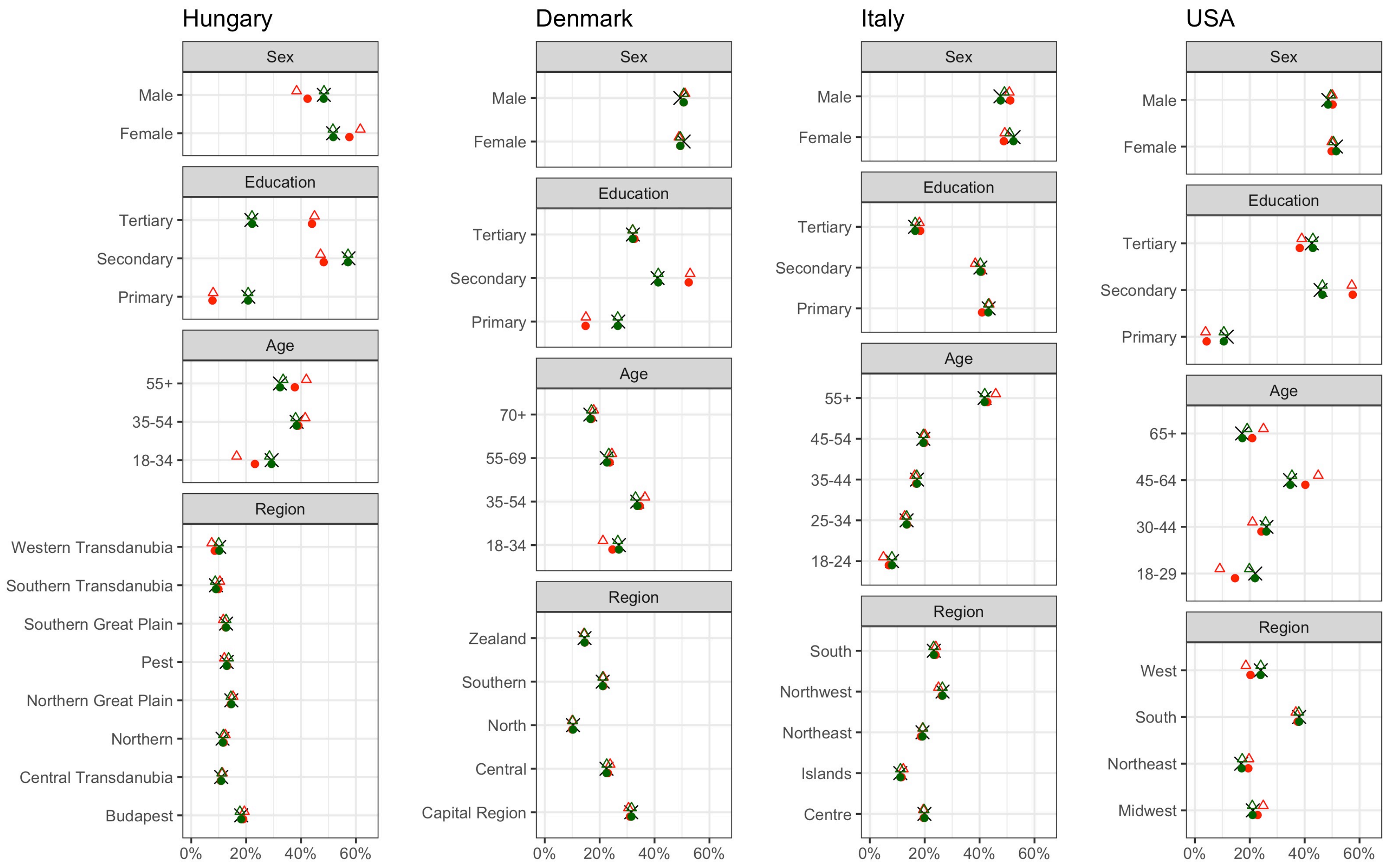

Figure OA1: Proportion of sex, education, age and region in four samples and four countries. Red marks denote unweighted samples, green marks denote weighted samples. Dots denote the full sample, triangles denote the balanced panel sample (i.e. only those who participated in all 3 waves). The large black cross denotes the population margins.

B.3 Descriptive statistics for our outcomes

Table OA2: Descriptive statistics in Denmark and Hungary for each of the three waves

	Denmark_1	Denmark_2	Denmark_3	Hungary_1	Hungary_2	Hungary_3
Subjective Burden of COVID-19	0.36 (0.14)	0.34 (0.14)	0.36 (0.14)	0.5 (0.14)	0.46 (0.16)	0.51 (0.14)
Anomie	0.36 (0.18)	0.35 (0.18)	0.36 (0.17)	0.44 (0.21)	0.41 (0.21)	0.44 (0.20)
Support for redistribution	0.44 (0.20)	0.41 (0.19)	0.42 (0.19)	0.52 (0.19)	0.53 (0.19)	0.53 (0.18)
Tolerate immigrants	0.38 (0.21)	0.38 (0.21)	0.37 (0.21)	0.38 (0.20)	0.38 (0.21)	0.38 (0.20)
Social trust	0.69 (0.46)	0.68 (0.47)	0.69 (0.46)	0.3 (0.46)	0.34 (0.47)	0.33 (0.47)
Reject surveillance	0.58 (0.25)	0.58 (0.25)	0.57 (0.25)	0.7 (0.24)	0.71 (0.22)	0.71 (0.24)
Satisfaction with pol. system	0.67 (0.23)	0.66 (0.24)	0.64 (0.25)	0.34 (0.31)	0.33 (0.31)	0.3 (0.30)
Level of Democracy	0.72 (0.22)	0.7 (0.22)	0.69 (0.25)	0.36 (0.31)	0.36 (0.31)	0.34 (0.31)
Support for democracy	0.83 (0.20)	0.82 (0.20)	0.84 (0.19)	0.76 (0.20)	0.77 (0.20)	0.77 (0.19)
Proud citizen	0.77 (0.24)	0.76 (0.24)	0.74 (0.24)	0.65 (0.29)	0.64 (0.30)	0.62 (0.30)
Need for chaos	0.17 (0.17)	0.18 (0.18)	0.16 (0.17)	0.23 (0.21)	0.22 (0.18)	0.22 (0.18)
Believe and share misinfo	0.22 (0.25)	0.24 (0.25)	0.2 (0.24)	0.37 (0.29)	0.34 (0.27)	0.35 (0.28)
Populism	0.51 (0.10)	0.52 (0.10)	0.51 (0.10)	0.55 (0.13)	0.56 (0.13)	0.56 (0.13)
Support for the Government	0.68 (0.20)	0.65 (0.21)	0.61 (0.24)	0.45 (0.27)	0.44 (0.27)	0.41 (0.27)
Affective polarization	0.23 (0.25)	0.23 (0.25)	0.23 (0.25)	0.25 (0.27)	0.26 (0.28)	0.28 (0.29)
Financial prospects	0.58 (0.16)	0.58 (0.16)	0.58 (0.16)	0.5 (0.19)	0.5 (0.20)	0.48 (0.19)

Table OA3: Descriptive statistics in Italy and the USA for each of the three waves

	Italy_1	Italy_2	Italy_3	USA_1	USA_2	USA_3
Subjective Burden of COVID-19	0.48 (0.13)	0.45 (0.14)	0.49 (0.13)	0.41 (0.13)	0.4 (0.14)	0.43 (0.14)
Anomie	0.48 (0.19)	0.46 (0.18)	0.48 (0.19)	0.34 (0.20)	0.34 (0.19)	0.34 (0.21)
Support for redistribution	0.45 (0.19)	0.45 (0.19)	0.44 (0.18)	0.42 (0.25)	0.41 (0.27)	0.41 (0.26)
Tolerate immigrants	0.42 (0.22)	0.42 (0.22)	0.43 (0.22)	0.5 (0.25)	0.51 (0.26)	0.52 (0.25)
Social trust	0.3 (0.46)	0.29 (0.46)	0.3 (0.46)	0.44 (0.50)	0.46 (0.50)	0.46 (0.50)
Reject surveillance	0.59 (0.26)	0.6 (0.27)	0.6 (0.25)	0.71 (0.26)	0.69 (0.26)	0.69 (0.27)
Satisfaction with pol. system	0.44 (0.28)	0.41 (0.27)	0.39 (0.27)	0.29 (0.29)	0.26 (0.28)	0.24 (0.27)
Level of Democracy	0.51 (0.29)	0.5 (0.29)	0.48 (0.30)	0.41 (0.28)	0.39 (0.28)	0.38 (0.27)
Support for democracy	0.71 (0.22)	0.72 (0.22)	0.72 (0.23)	0.79 (0.21)	0.79 (0.22)	0.79 (0.22)
Proud citizen	0.71 (0.28)	0.67 (0.28)	0.65 (0.29)	0.77 (0.30)	0.74 (0.32)	0.74 (0.30)
Need for chaos	0.26 (0.19)	0.26 (0.18)	0.28 (0.20)	0.2 (0.20)	0.19 (0.19)	0.19 (0.20)
Believe and share misinfo	0.41 (0.30)	0.39 (0.28)	0.4 (0.29)	0.34 (0.30)	0.34 (0.31)	0.34 (0.31)
Populism	0.59 (0.11)	0.58 (0.11)	0.58 (0.11)	0.61 (0.13)	0.62 (0.13)	0.62 (0.13)
Support for the Government	0.54 (0.20)	0.53 (0.20)	0.5 (0.21)	0.5 (0.20)	0.44 (0.19)	0.41 (0.18)
Affective polarization	0.28 (0.28)	0.29 (0.29)	0.26 (0.27)	0.38 (0.35)	0.44 (0.33)	0.4 (0.33)
Financial prospects	0.41 (0.20)	0.42 (0.20)	0.41 (0.19)	0.52 (0.22)	0.54 (0.22)	0.51 (0.21)

B.4 Test-retest correlations and alpha reliability estimates

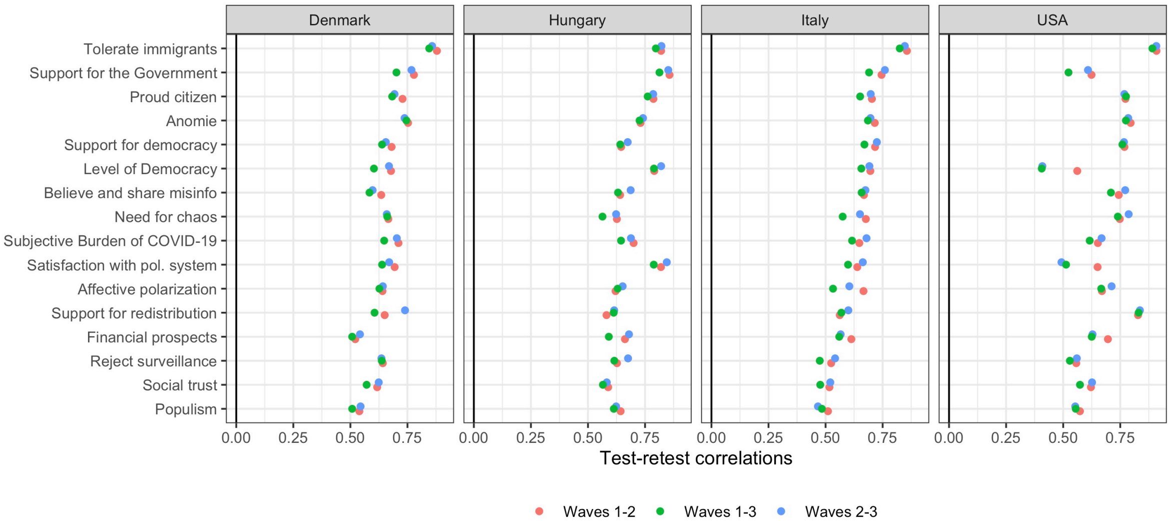

Figure OA2: Test-retest correlation estimates for our measures

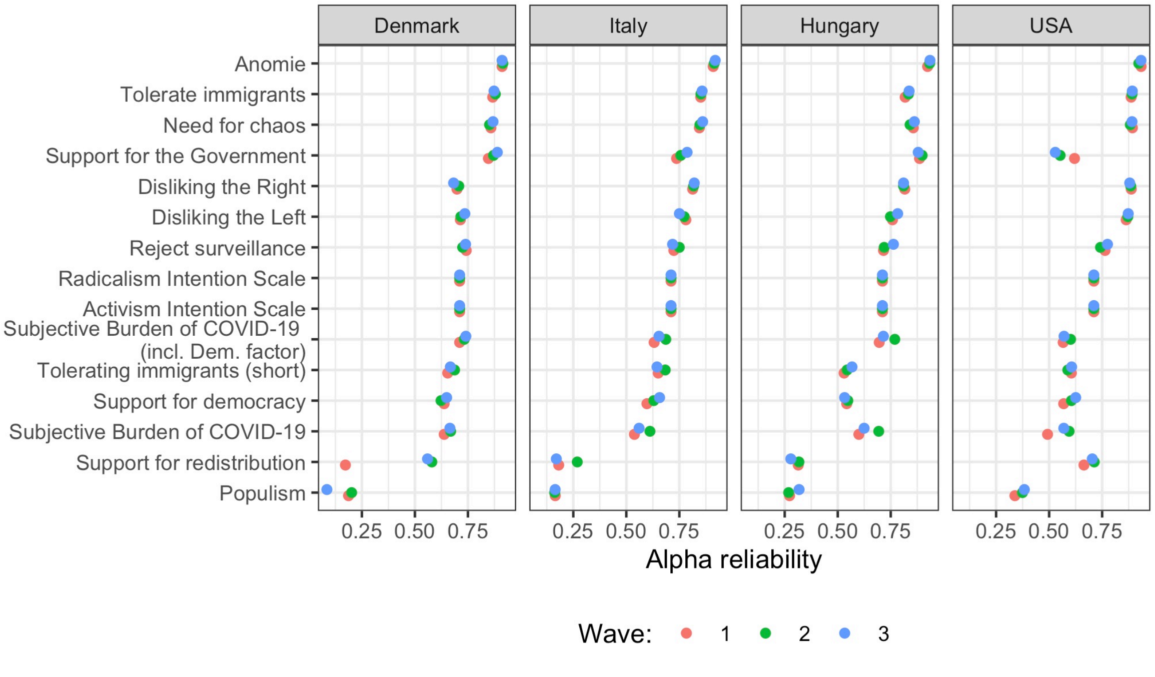

Figure OA3: Alpha reliability estimates for multi-item scales

C Model details and additional analyses

C.1 Confidence intervals and regression tables for Fig 1

To offer a more detailed overview of our time trends, Figures OA4 and OA5 reproduce 2, but also display all confidence intervals. Tables ?? - OA7 in turn display regressions contrasting the April data with June and December mean results. The main conclusion that the negative trend in system support is significant in three of the four variables (the only exception is support for democracy), and pooling across the variables, there is a significant negative change between April and December in all 4 countries. Meanwhile, all pooled changes in social solidarity are substantively small and not statistically significant at conventional levels.

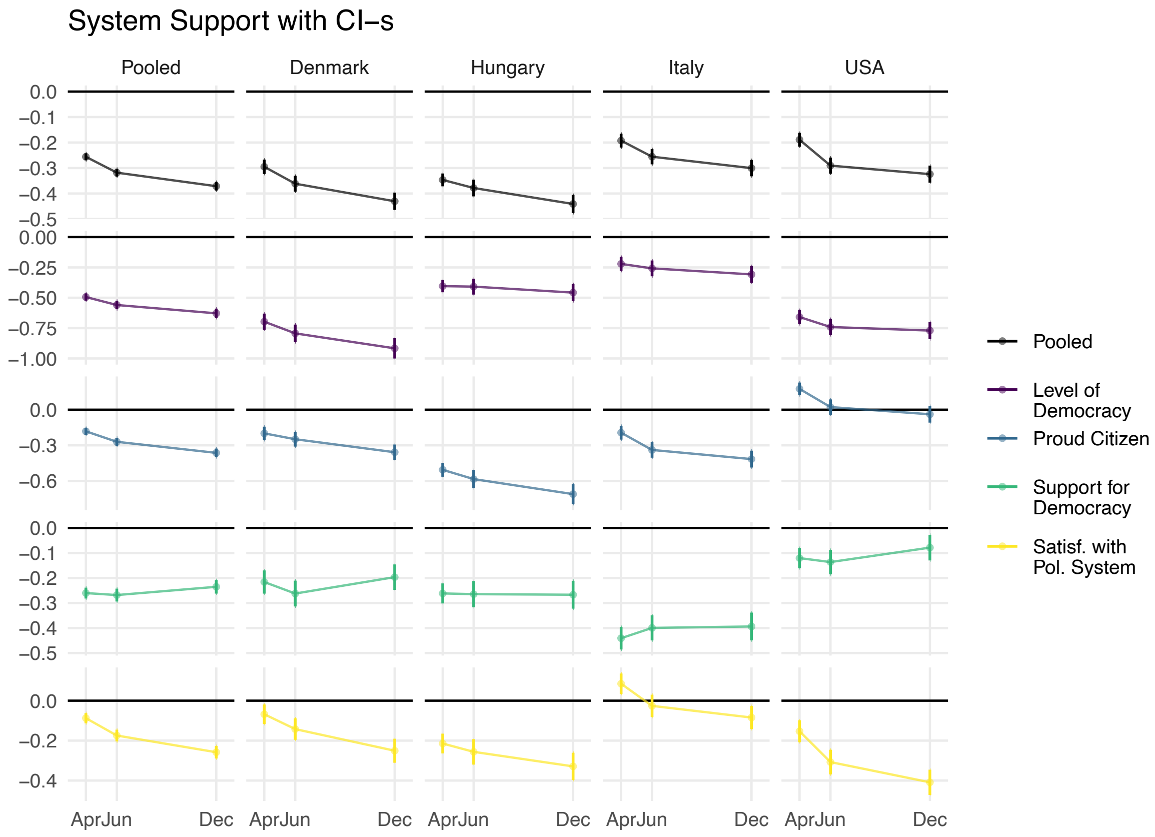

Figure OA4: Reproducing Fig 1. System Support subplot with all confidence intervals

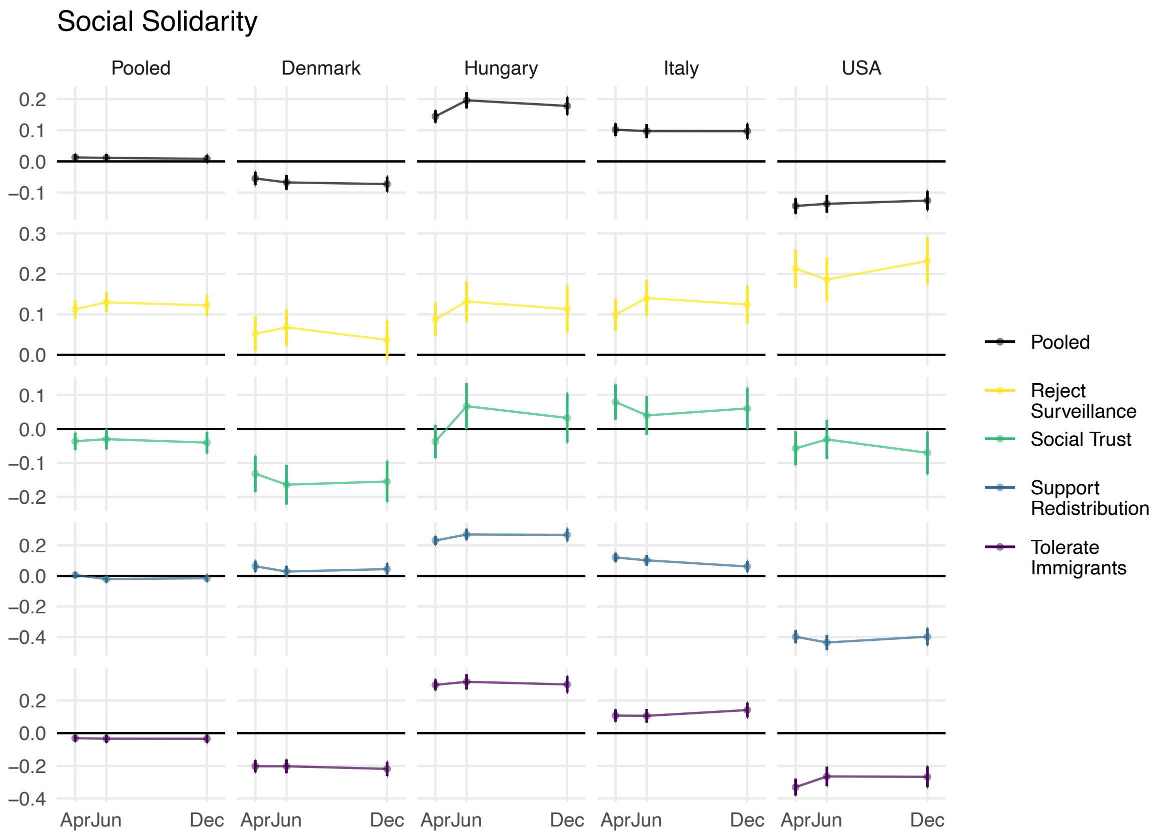

Figure OA5: Reproducing Fig 1. Social Solidarity subplot with all confidence intervals

Table OA4: Time trends system support variables pooling across countries

	Dependent variable:			
	Sys.stfctn	Level.of.demcy	Supp.demcy	Proud.ctzn
	(1)	(2)	(3)	(4)
Constant	−0.09***	−0.48***	−0.23***	−0.19***
	(0.01)	(0.02)	(0.01)	(0.01)
wave_2	−0.10***	−0.07**	0.01	−0.06*
	(0.02)	(0.02)	(0.02)	(0.02)
wave_3	−0.18***	−0.14***	0.06**	−0.11***
	(0.02)	(0.02)	(0.02)	(0.02)
Observations	14,717	14,717	14,717	14,717
Adjusted R^2	0.005	0.002	0.001	0.001
Note:				*p<0.05; **p<0.01; ***p<0.001

Table OA5: Time trends in pooled system support across countries

	Dependent variable:			
	Pooled System Support			
	Denmark	Hungary	Italy	USA
	(1)	(2)	(3)	(4)
Constant	−0.27***	−0.33***	−0.18***	−0.20***
	(0.02)	(0.02)	(0.02)	(0.01)
wave_2	−0.06*	−0.01	−0.07**	−0.08***
	(0.03)	(0.03)	(0.03)	(0.02)
wave_3	−0.10**	−0.06*	−0.11***	−0.11***
	(0.03)	(0.03)	(0.03)	(0.02)
Observations	4,000	3,193	3,881	3,643
Adjusted R^2	0.002	0.001	0.004	0.01
Note:				*p<0.05; **p<0.01; ***p<0.001

Table OA6: Time trends social solidarity variables pooling across countries

	Dependent variable:			
	Soc.solid	Toler.immig	Soc.trust	No.surveillance
	(1)	(2)	(3)	(4)
Constant	−0.01	−0.02	−0.01	0.13***
	(0.01)	(0.01)	(0.01)	(0.01)
wave_2	−0.04**	−0.02	0.0000	0.02
	(0.01)	(0.02)	(0.02)	(0.02)
wave_3	−0.04**	−0.03	0.01	−0.0002
	(0.02)	(0.02)	(0.02)	(0.02)
Observations	14,717	14,717	14,717	14,717
Adjusted R^2	0.001	0.0001	−0.0001	−0.0000
Note:		*p<0.05; **p<0.01; ***p<0.001		

Table OA7: Time trends in pooled social solidarity across countries

	Dependent variable:			
	Pooled Social Solidarity			
	Denmark	Hungary	Italy	USA
	(1)	(2)	(3)	(4)
Constant	−0.04**	0.17***	0.11***	−0.15***
	(0.01)	(0.01)	(0.01)	(0.01)
wave_2	−0.02	0.04*	−0.01	−0.004
	(0.02)	(0.02)	(0.02)	(0.02)
wave_3	−0.02	0.03	−0.02	−0.01
	(0.02)	(0.02)	(0.02)	(0.02)
Observations	4,000	3,193	3,881	3,643
Adjusted R^2	−0.0001	0.001	−0.0003	−0.001
Note:		*p<0.05; **p<0.01; ***p<0.001		

C.2 Regression tables accompanying Fig 3

Table OA8: Fixed effects regressions of social solidarity on burden of COVID-19

	Dependent variable:				
	Pooled	Supp for Redistr	Toler Immig	Soc Trst	Reject Surv
	(1)	(2)	(3)	(4)	(5)
Covid Burden	−0.0001	0.01***	−0.01*	−0.01	0.01
	(0.01)	(0.005)	(0.003)	(0.01)	(0.01)
Observations	43,440	10,860	10,860	10,860	10,860
Adjusted R^2	0.16	0.70	0.87	0.61	0.59
Note:					*p<0.1; **p<0.05; ***p<0.01

Table OA9: Fixed effects regressions of social solidarity on anomie

	Dependent variable:				
	Pooled	Supp for Redistr	Toler Immig	Soc Trst	Reject Surv
	(1)	(2)	(3)	(4)	(5)
Anomie	−0.005	0.01***	−0.01***	−0.01	−0.02***
	(0.01)	(0.004)	(0.003)	(0.01)	(0.01)
Observations	43,440	10,860	10,860	10,860	10,860
Adjusted R^2	0.16	0.70	0.87	0.61	0.59
Note:					*p<0.1; **p<0.05; ***p<0.01

Table OA10: Fixed effects regressions of system support on burden of COVID-19

	Dependent variable:				
	Pooled	System Stsf.	Lvl of Demcy	Supp Demcy	Proud Cit
	(1)	(2)	(3)	(4)	(5)
Covid Burden	−0.04***	−0.05***	−0.04***	−0.02***	−0.03***
	(0.01)	(0.01)	(0.01)	(0.005)	(0.01)
Observations	43,440	10,860	10,860	10,860	10,860
Adjusted R^2	0.25	0.74	0.71	0.72	0.73
Note:					*p<0.1; **p<0.05; ***p<0.01

Table OA11: Fixed effects regressions of system support on Anomie

	Dependent variable:				
	Pooled	System Stsf.	Lvl of Demcy	Supp Demcy	Proud Cit
	(1)	(2)	(3)	(4)	(5)
Anomie	−0.03***	−0.03***	−0.03***	−0.02***	−0.03***
	(0.005)	(0.01)	(0.01)	(0.004)	(0.01)
Observations	43,440	10,860	10,860	10,860	10,860
Adjusted R^2	0.25	0.74	0.71	0.72	0.73
Note:					*p<0.1; **p<0.05; ***p<0.01

Table OA12: Fixed effects regressions of extreme discontent on burden of COVID-19

	Dependent variable:			
	Pooled	Need for Chaos	Misinfo	Populism
	(1)	(2)	(3)	(4)
Covid Burden	0.02***	0.03***	0.03***	0.01***
	(0.01)	(0.004)	(0.01)	(0.003)
Observations	32,580	10,860	10,860	10,860
Adjusted R^2	0.18	0.69	0.69	0.60
Note:				*p<0.1; **p<0.05; ***p<0.01

Table OA13: Fixed effects regressions of extreme discontent on anomie

	Dependent variable:			
	Pooled	Need for Chaos	Misinfo	Populism
	(1)	(2)	(3)	(4)
Anomie	0.04***	0.07***	0.04***	0.01***
	(0.005)	(0.004)	(0.01)	(0.003)
Observations	32,580	10,860	10,860	10,860
Adjusted R^2	0.18	0.70	0.69	0.60
Note:				*p<0.1; **p<0.05; ***p<0.01

C.3 Which demographic groups experience more burden and anomie during 2020?

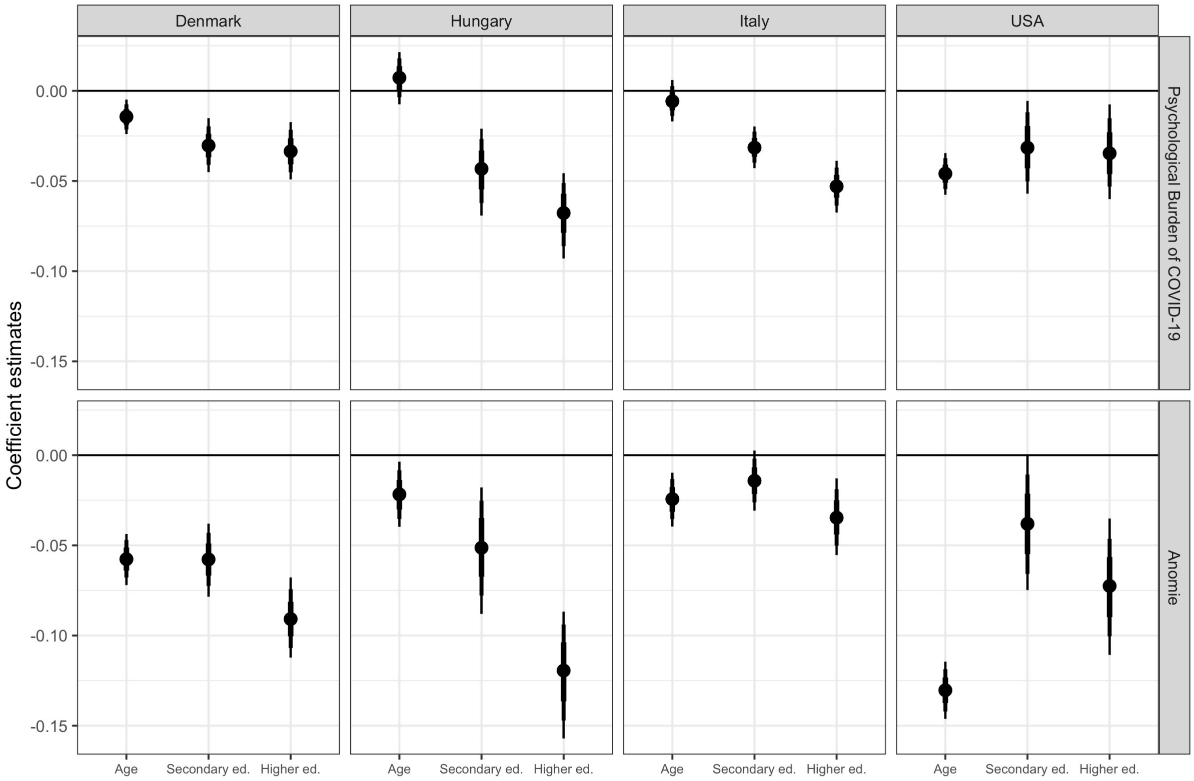

Figure OA6: Older and more highly educated respondents perceived less covid burden and anomie

To better understand who experienced a higher subjective burden from the COVID-19 crisis, we perform to sets of analyses: within-respondent and cross-sectional models. First, we regress covid burden and anomie on three indicators of the pandemic's immediate effects: perceptions of own physical health, getting sick with COVID-19 and getting unemployed. We find that when respondents report a decrease in physical health, get sick with COVID-19 or lose their jobs, they also report slightly higher covid burden. Admittedly, these effects are small amounting to only a 0.01-0.03 change in burden. Moreover, only 9% of respondents reported getting sick with COVID-19[9] and only 12% of respondents had a change in their employment status throughout our study period, therefore these relationships are driven by small subsamples. We also find that a decrease in physical health is associated to a small increase in anomie, but getting COVID-19 or (un)employed is not associated to changes in anomie. All in all, these analyses show that the subjective covid burden is influenced, but is not driven by dramatic changes in personal health or employment. This conclusion is consistent with the aggregate analyses which show relatively little responsiveness to dramatic changes in epidemic trajectories.

We also investigated cross-sectional differences in covid burden and anomie. Specifically, we built multilevel regression models regressing burden and anomie on age, gender and level of education. We investigated heterogeneities across countries and waves by investigating if varying intercepts and slopes improve model fit. We found differences in levels of burden and anomie across both time and countries. We found that the demographic differences for age and education vary with country, but remain stable across time. Finally, gender has consistently no effect on burden and anomie.

[9]As our question asks if the respondent was ever sick with COVID-19, we exclude respondents, who report COVID-19 but at a later wave renege on it. Thereby, we restrict the analysis to pre- post-comparisons of personal experience with the virus.

Figure OA6 displays the coefficient estimates for secondary and higher education, as well as age for both burden and anomie in each of the four countries. To make the estimates comparable, we standardize the age variable by centering and dividing by two standard deviations. We find that on average younger respondents perceive slightly more burden (b = 0.01) and higher anomie (b = 0.06). This is surprising given that older people were much more likely to get seriously ill from the virus, but may reflect the fact that for most people the corona-crisis was not primarily about their physical health. We also find that higher educational attainment appears to shield from both burden and anomie. Compared to those with only a primary education, respondents with a secondary (burden b = -0.03, anomie b = -0.04) and especially a tertiary education (burden b = -0.05, anomie b = -0.08) are less negatively affected by the pandemic.

C.4 Estimating realistic changes in our predictors

In order to estimate a realistic within-unit change in our two predictors, we followed the following procedure: First, we regressed both predictors on the two fixed effects (respondent and country-wave). Then we exported the residuals from these models or in other words the demeaned predictors employed in our analyses. Next, for each individual, we calculated the largest difference between any two waves. Figure OA7 below depict the distribution of these estimates.

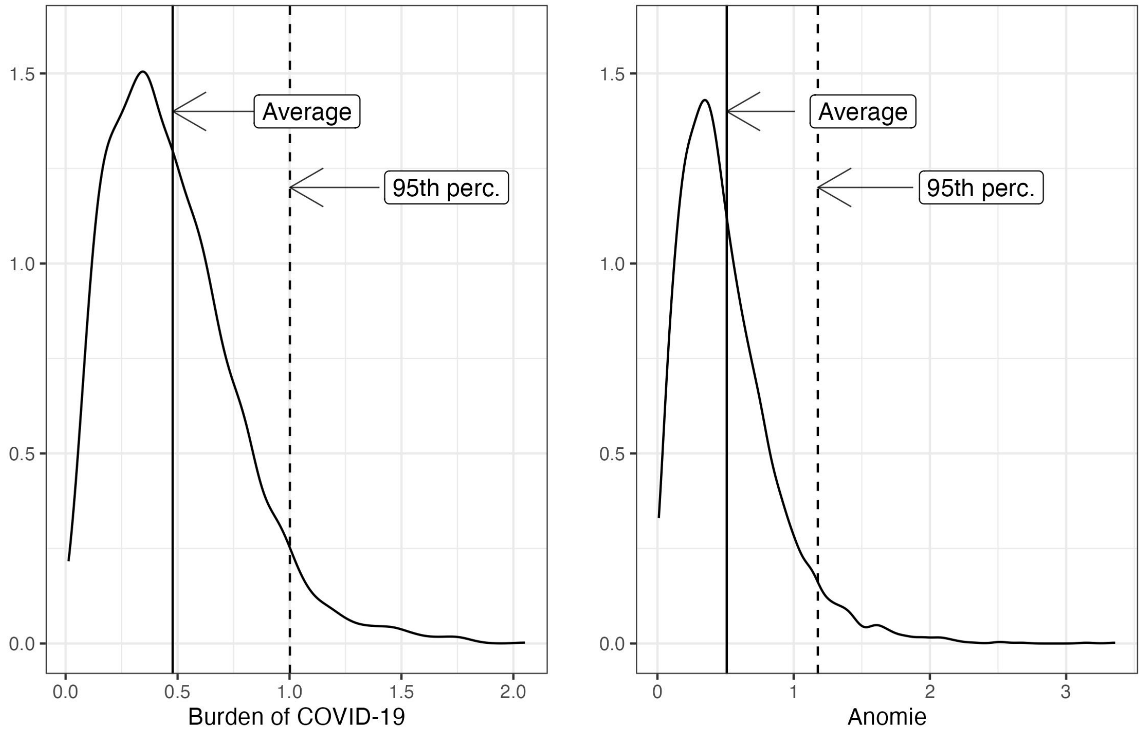

Figure OA7: Distribution of within-unit changes in our predictors. Horizontal lines mark the average (black line) and a large but still realistic (dashed line) within-unit change in the covid burden and Anomie.

C.5 Descriptive Trends in Extreme Discontent

While we are unable to benchmark the levels of extreme discontent measured, we can still inspect their changes throughout the pandemic. Figure OA8 demonstrate that for the most part, average levels of extreme discontent were stable throughout the study period. We find the highest levels of extreme discontent in Italy and Hungary, with a faint pattern resembling the changes in the pandemic burden. Extreme discontent was lower in the US, and has been slightly but steadily increasing between April

and December. Unsurprisingly, the lowest levels of extreme discontent were found in Denmark, but surprisingly, the within country trends moved oppose to the pandemic with the highest value observed in June.

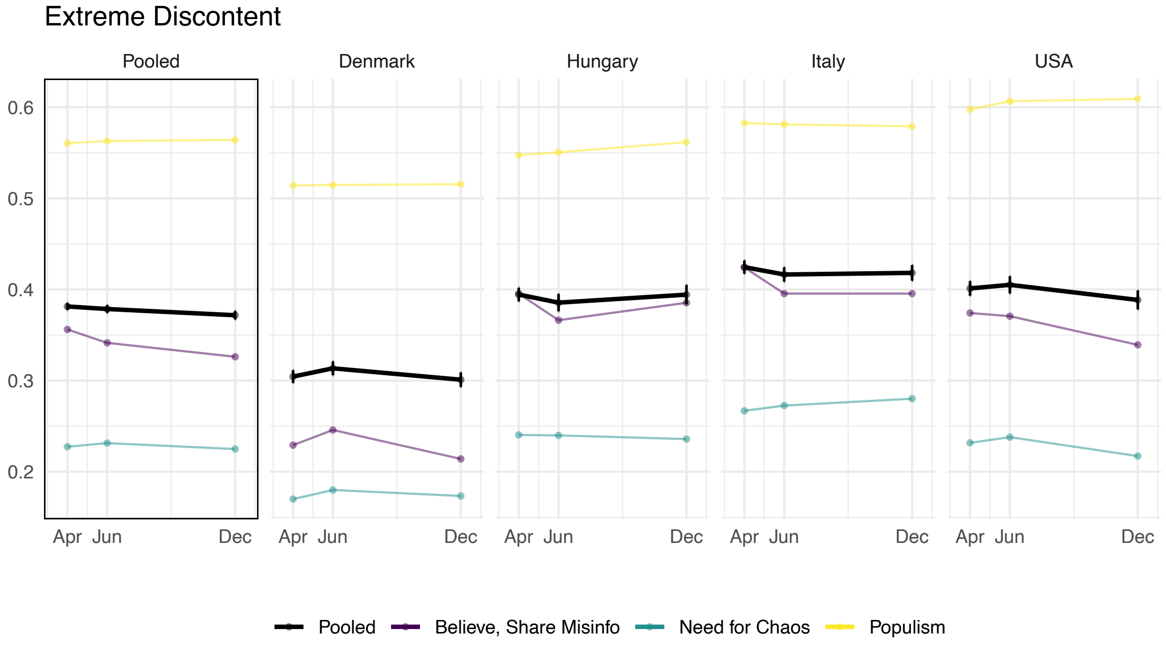

Figure OA8: Descriptive trends in extreme discontent.

C.6 Cross-country variation in 2FE models

Regarding social solidarity we observe two notable heterogeneities in Figure OA9. First, while we reported a tiny overall increase in support for redistribution for changes in covid burden, it appears that this masks larger positive relationships in Hungary, Italy and the US, tempered by a negative relationship in Denmark. Second, we found that an increase in Anomie is associated with a decrease in the rejection of surveillance. Our country-wise models show that this relationship is entirely driven by a large association among Hungarians, tempered by no relationships in the other three countries.

Next, we consider the category of System Support. Although the pooled country-wise estimates are not significantly different from each other, the US estimate appears to be consistently smaller than the ones in the other countries, driven primarily by negligible associations between covid burden and level of democracy, and support for the political system. It is notable that changes in covid burden do not predict changes in perceived levels of democracy in Hungary either, which happens to be the other country in our sample experiencing democratic backsliding. Other than these, all associations – and notably all associations with change in Anomie – appear to be highly consistent across countries.

Finally, we consider Extreme Discontent. Overall, the heterogeneities are smaller here than with System Support. The pooled relationship between covid burden and Extreme Discontent is somewhat smaller in Hungary and the US than in Italy and Denmark. If anything, changes in believing and sharing misinformation are negatively related to changes in burden in Hungary, although we find the expected positive associations in the other three countries. Again, it is notable that all relationships between anomie and various types of extreme discontent are remarkably consistent across the four countries.

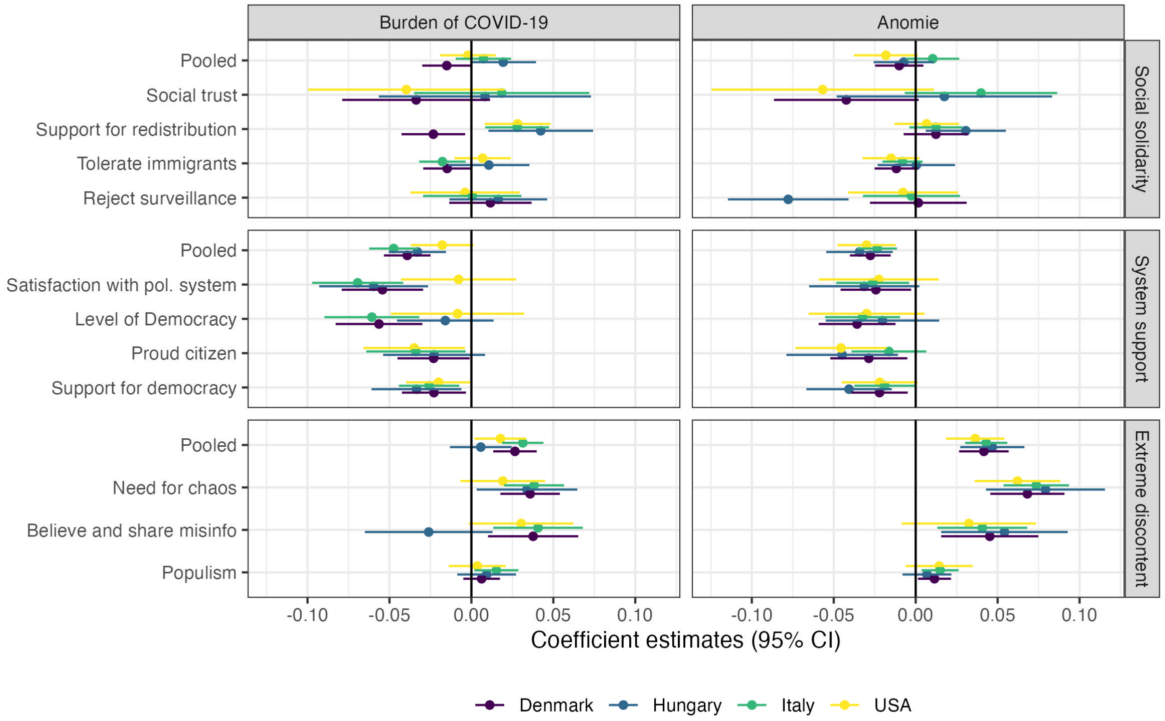

Figure OA9: 2FE models split by the four countries.

C.7 Additional outcome variables

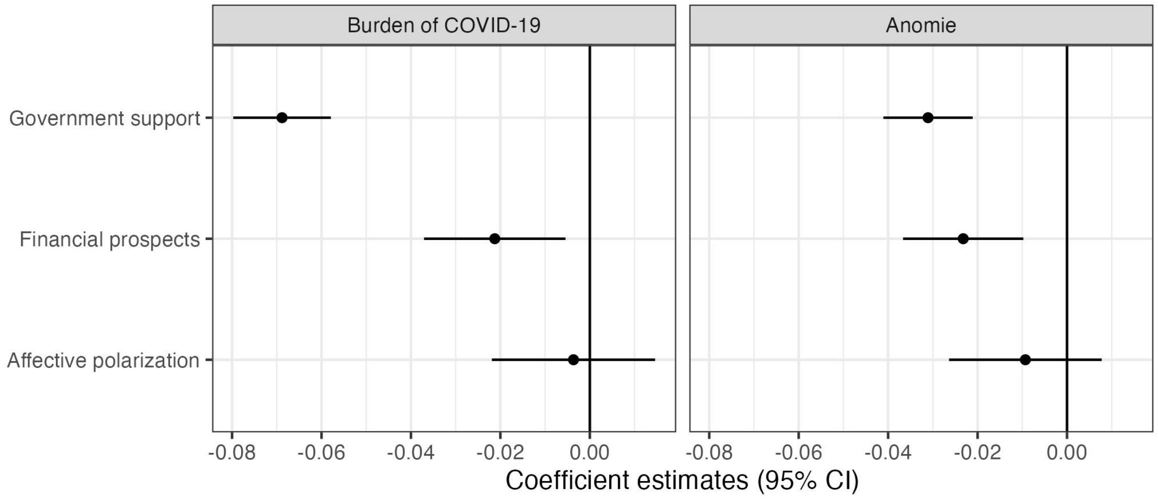

Figure OA10: Additional outcome variables

C.8 Splitting Covid Burden into component factors

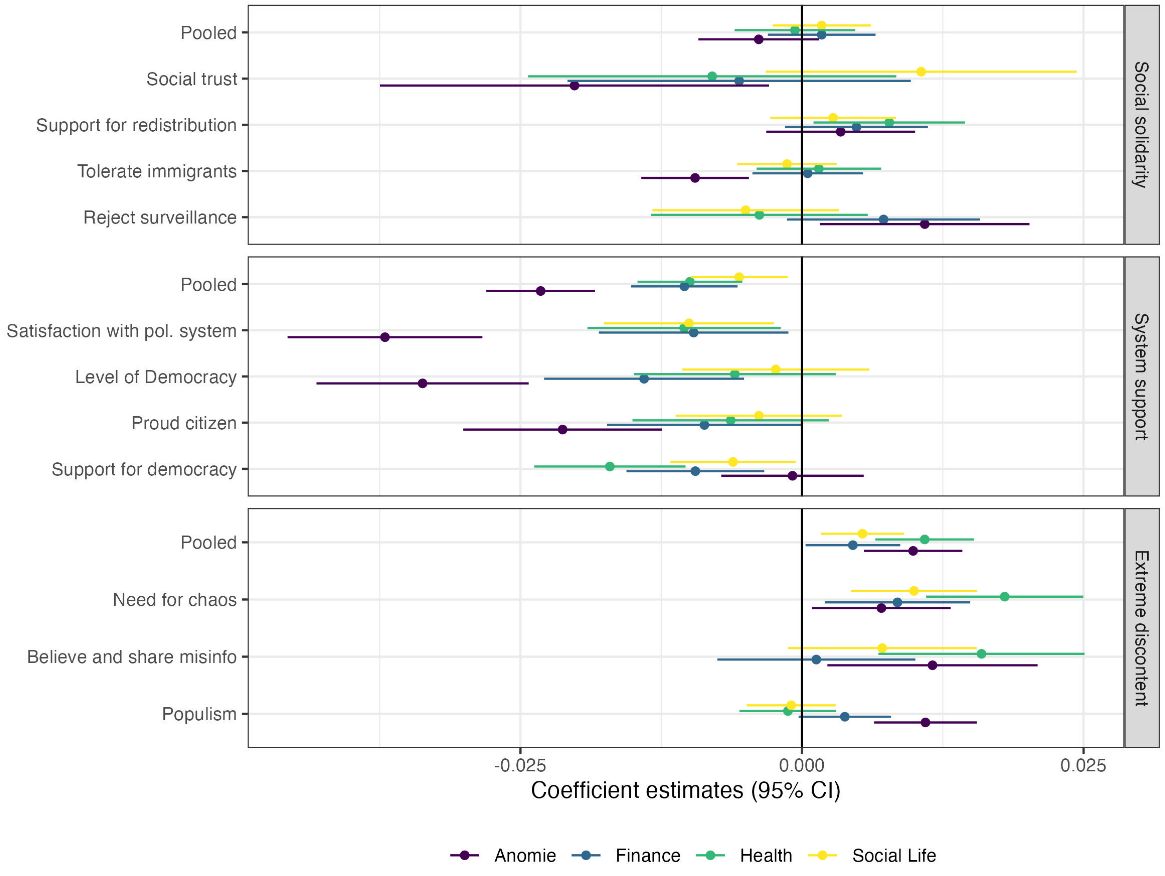

Figure OA11: Splitting COVID burden into component factors

C.9 COVID-19 Burden, Anomie and Antigovernment Behavior Intentions – Replicating and extending Bartusevicius et al 2021.

Bartusevičius and colleagues [19] provided evidence that respondents who suffered from a higher subjective COVID-19 burden at Wave 1 of data collection were also more likely to indicate an intention to participate in both peaceful and violent protests at Wave 2. These analyses relied on validated measures of activism intentions.[10] We replicate and extend these analyses by conducting two-way fixed effects models relying on Wave 2 and 3 data (the only two waves where behavioral intentions were measured). Table OA14 below reports these coefficient estimates. These models show that while the relationships between COVID-19 burden, anomie and (peaceful) activism intentions are generally weak, both changes in burden and in anomie are significantly associated to changes in (violenet) radicalism intentions.

Table OA14: 2FE models of Activism and Radicalism Intentions

	Dependent variable:			
	Activism Intentions		Radicalism Intentions	
	(1)	(2)	(3)	(4)
Covid-19 Burden	0.013		0.027**	
	(0.012)		(0.011)	
Anomie		0.019*		0.032***
		(0.010)		(0.010)
Observations	6,860	6,860	6,869	6,869
Adjusted R^2	0.565	0.566	0.538	0.539
Note:				*p<0.1; **p<0.05; ***p<0.01

[10] Moskalenko, S., & McCauley, C. (2009). Measuring political mobilization: The distinction between activism and radicalism. Terrorism and political violence, 21(2), 239-260.

C.10 The benchmarked, single-item measure of anomie

Our survey included an alternative, single-item measure of anomie, which we could benchmark to EVS and WVS data. The question read: "Some people feel they have completely free choice and control over their lives, while other people feel that what they do has no real effect on what happens to them. Please use this scale where 1 means "no choice at all" and 10 means "a great deal of choice" to indicate how much freedom of choice and control you feel you have over the way your life turns out." Figure OA13 below reports z-scored averages for this item in each country. We find that in our survey, people on average reported much higher levels of anomie than compared to the benchmarked surveys. Across the four countries, this difference is 35% of a standard deviation, ranging from 30% in the USA, to above 40% in Italy. We also find that variation in the average levels of anomie during the study period (and the COVID-19 pandemic) is much smaller than the initial change.

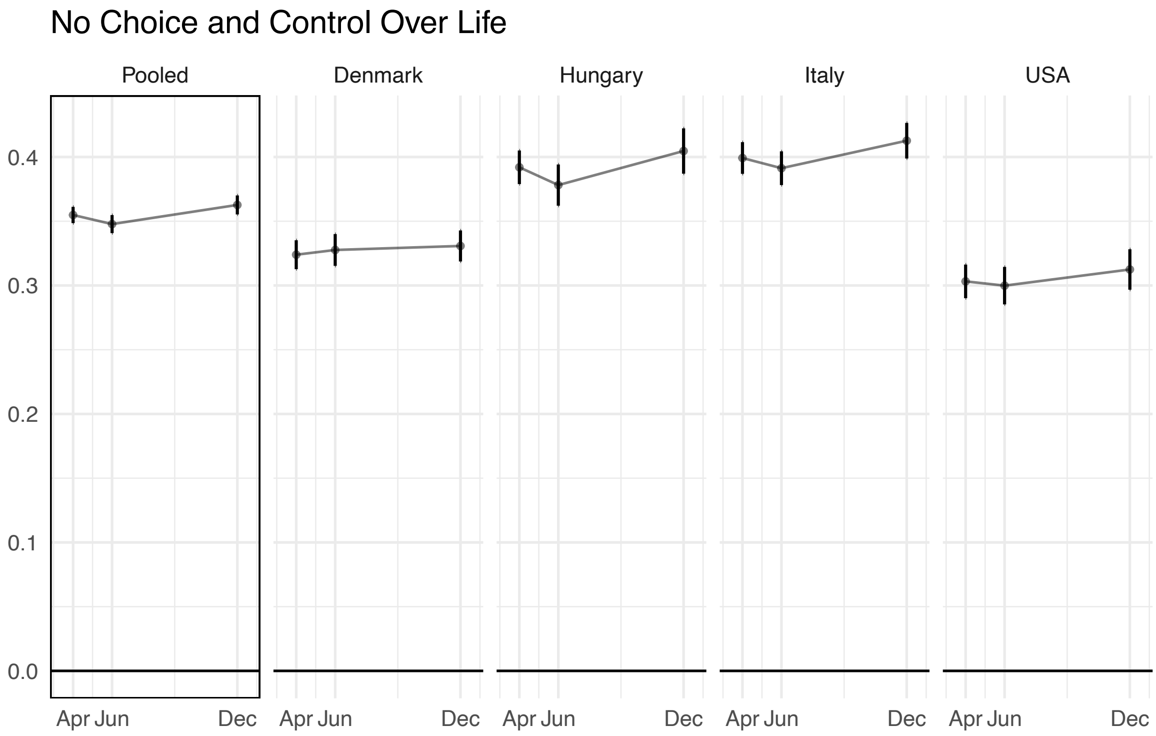

Figure OA12: Benchmarked changes in an alternative measure of anomie: across the four countries, people on average felt about 35% of a standard deviation more anomie during the pandemic than before it.

Figure OA13 below demonstrates that the single item measure of anomie replicates almost all two-way fixed effects estimates from our main analyses, with the caveat that as one would expect, these estimates are less precise, presumably due to higher measurement error. In practice means that the associations with system support variables are systematically over-estimated, whereas associations with extreme discontent are systematically under-estimated.

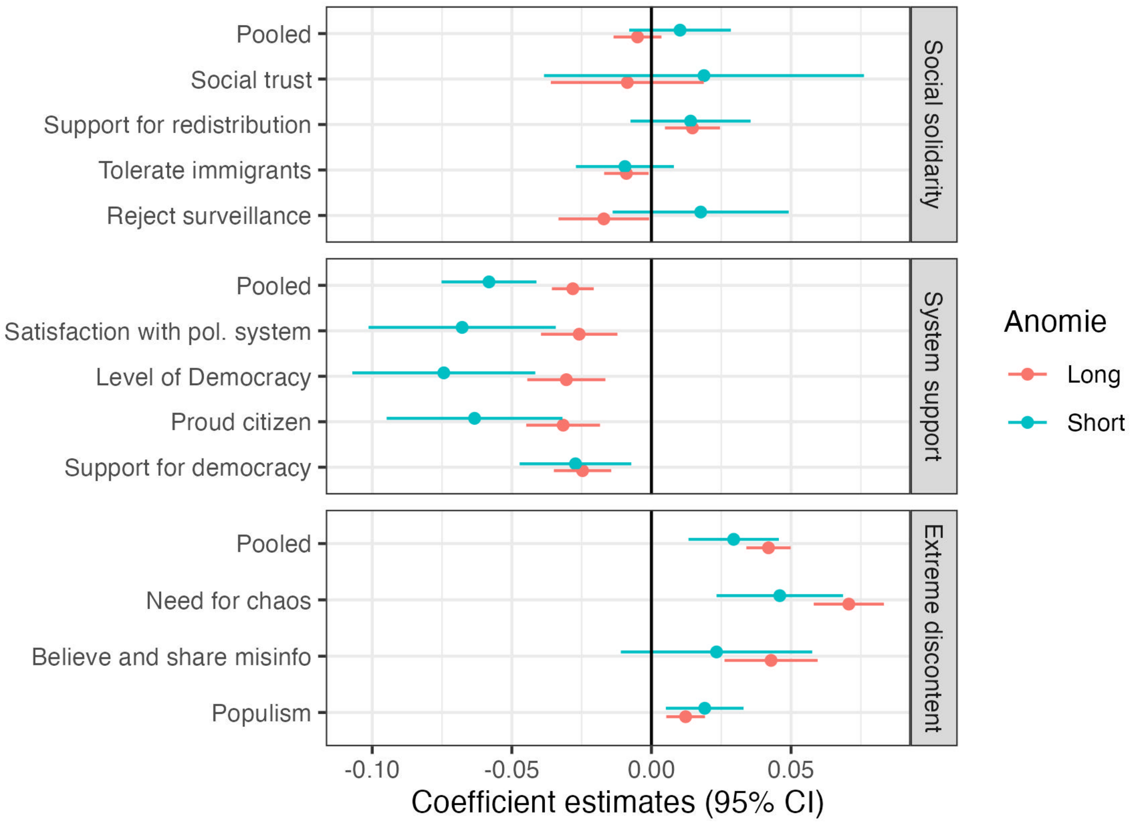

Figure OA13: Comparing original 2FE estimates of anomie to a single-item anomie measure, which we could benchmark to pre-pandemic levels

D Robustness checks

D.1 Full CBS scale

Originally, we considered a democratic burden to be part of the many effects of the pandemic on citizens. Upon reflection and feedback from our peers, we concluded that these evaluations may be endogenous to some of our outcomes – particularly when it comes to system support. Therefore, our main analyses did not include this factor. Yet, in the name of transparency, Figure OA14 below contrasts 2FE estimates with and without this facet ouf our COVID-19 burden scale.

As one would expect, the within-individual associations between COVID-burden are a bit stronger when it comes to system support and extreme discontent if burden also involves democratic aspects. These results are consistent with the possibility that besides the perceived burdens in finance, health, social life and anomie, concerns over democratic rights and freedoms have also contributed to the waning system support throughout the pandemic.

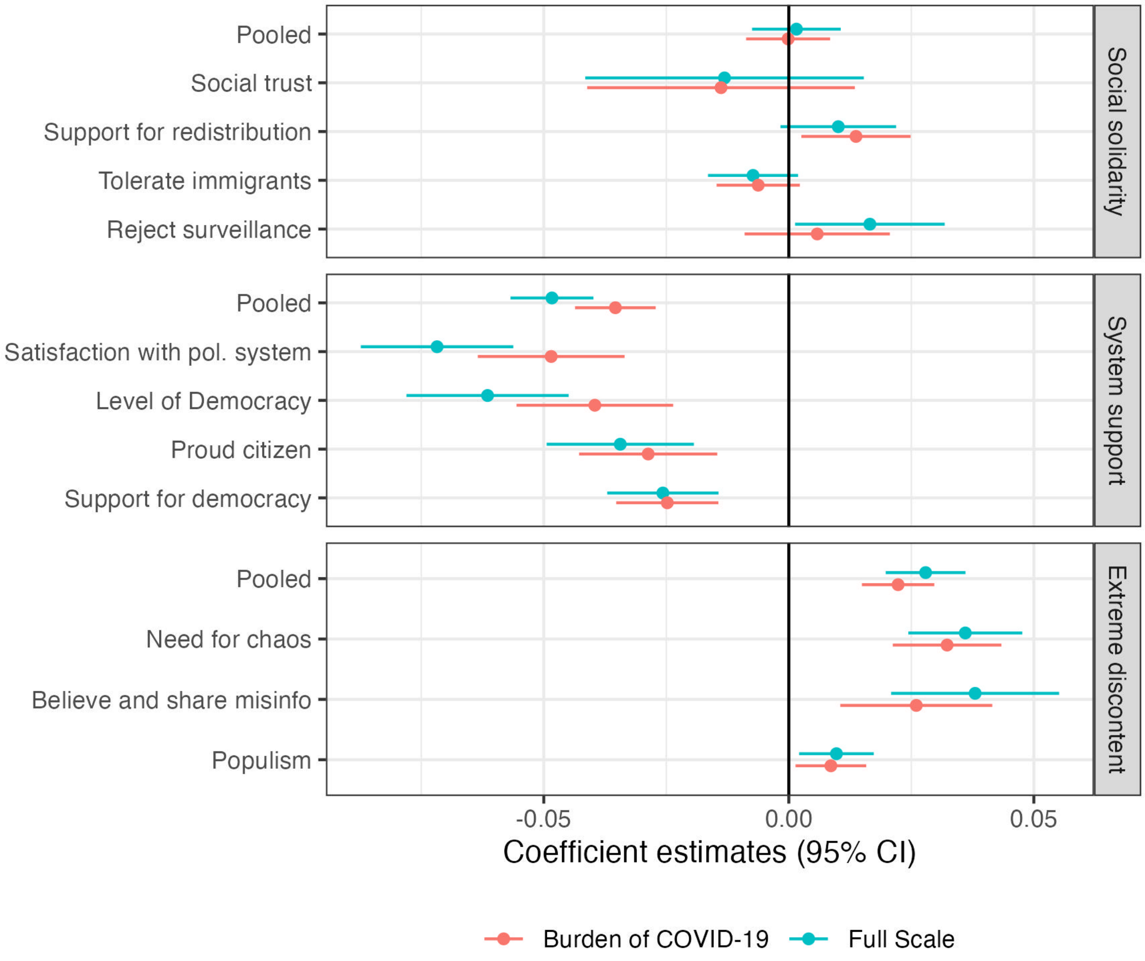

Figure OA14: Comparing original 2FE estimates with the full COVID-19 burden scale which includes the fifth component tapping into concerns about democracy

D.2 Testing the parallel trends assumption

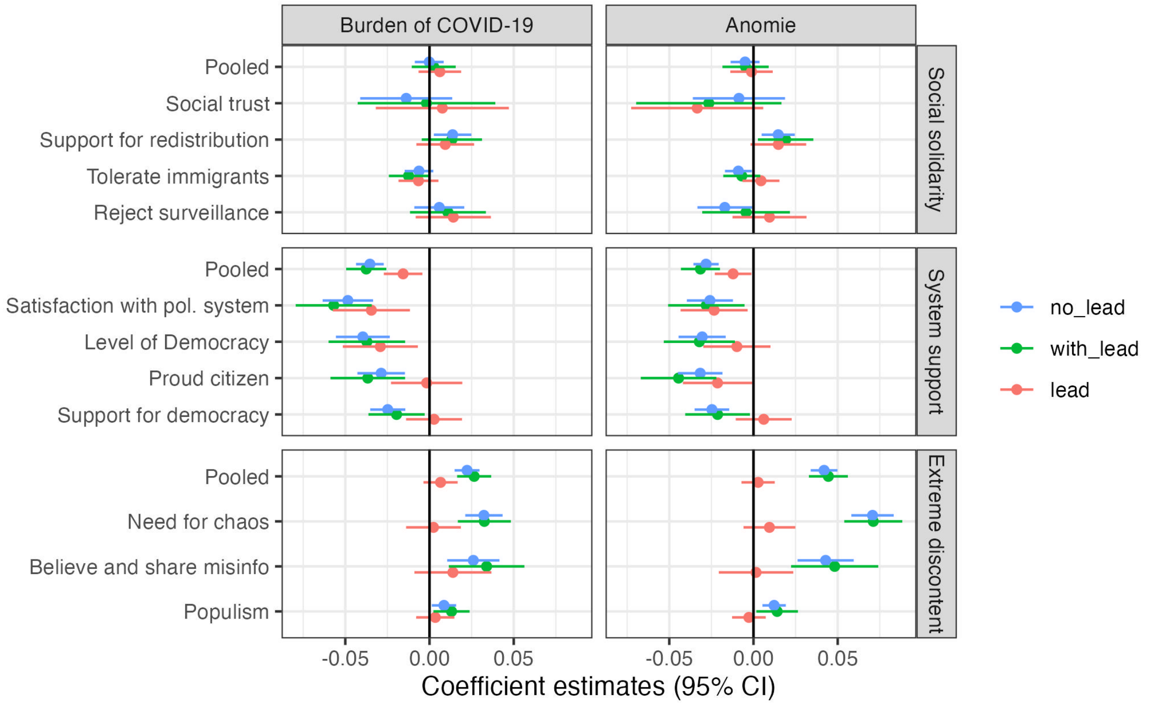

Figure OA15: Comparing original 2FE estimates with models that also include a leaded predictor variable

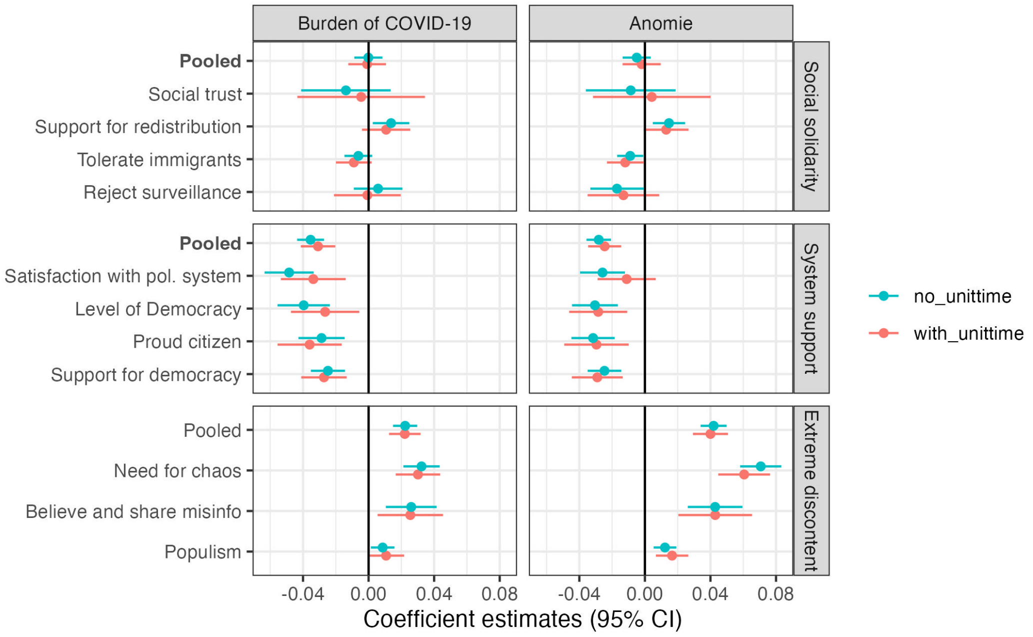

Figure OA16: Comparing original 2FE estimates with models that also include respondent-specific linear time trends

D.3 Controlling for Support for government

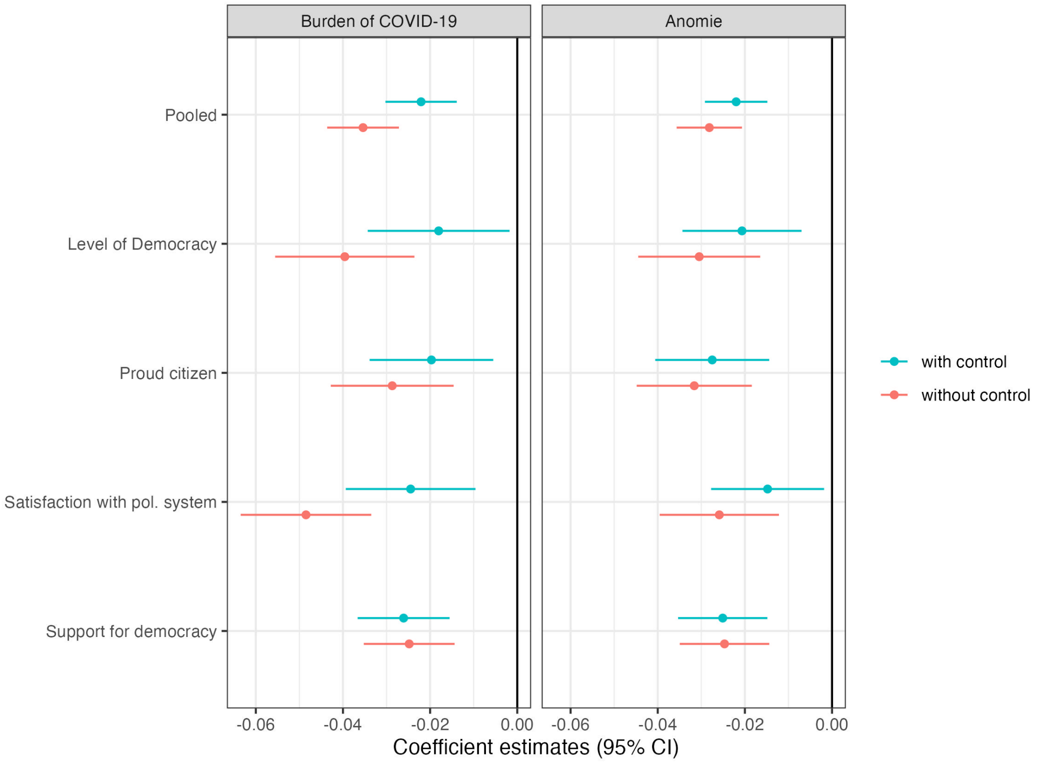

Figure OA17: Comparing original 2FE estimates for system support variables with models that adjust for support for the government

D.4 Comparing one and two-way fixed effects models

D.5 Replicating models without weights

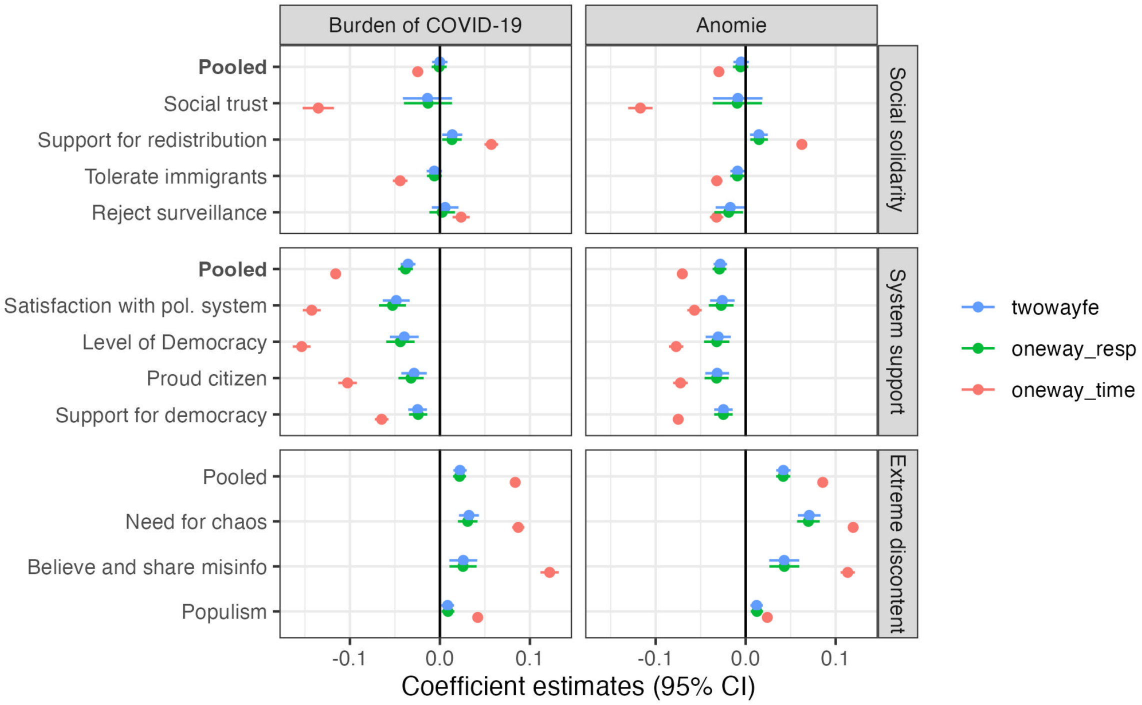

Figure OA18: Comparing original 2FE estimates with unit-fixed effects and time-fixed effects model estimates

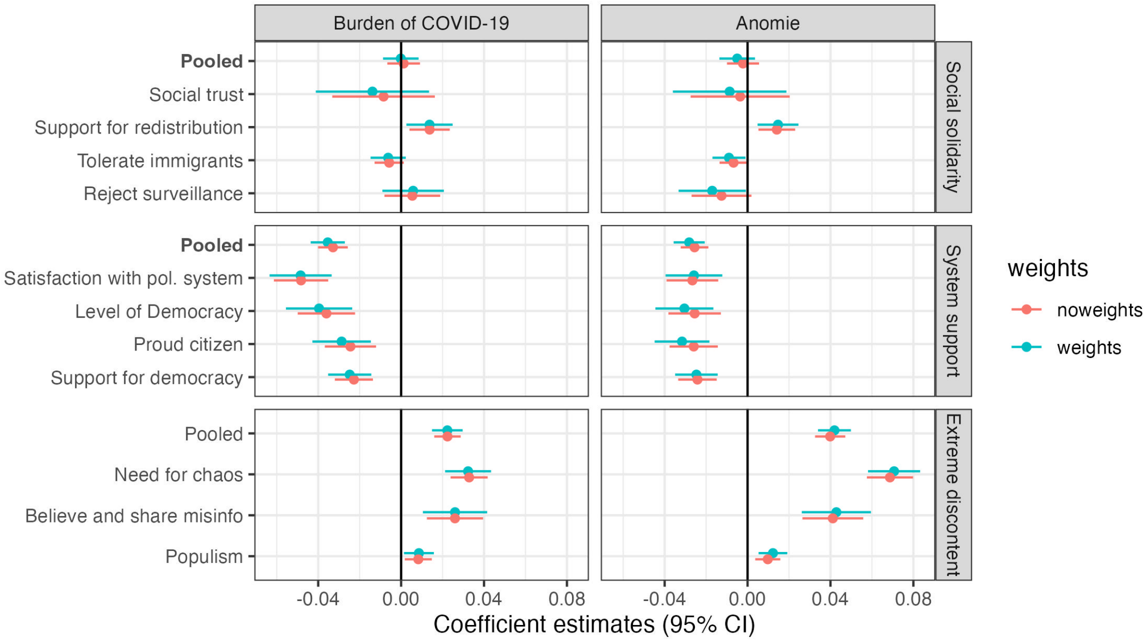

Figure OA19: Comparing original 2FE estimates with models without survey weights
