# Supplementary material for: The COVID-19 pandemic eroded system support but not social solidarity
Source: PLoS One. 2023 Aug 17;18(8):e0288644. doi: 10.1371/journal.pone.0288644 (PMC10434936; doi:10.1371/journal.pone.0288644)
Supplement: Attachment — Submitted filename: Undemocracy Memo RnR.docx [file pone.0288644.s002.docx]

## Reviewer #1

I reviewed the paper “The COVID-19 Pandemic Eroded System Support but Not Social Solidarity.” In this paper, the authors report the results of a panel survey conducted in Denmark, Hungary, Italy, and the US over the course of 2020 as the pandemic unfolded. They show that, over three survey waves, respondents are increasingly less satisfied with the way political institutions/democracy work/s in their country (contrary to preferences for redistribution and attitudes towards migration that did not change much). Then, they show that such satisfaction is negatively associated with subjective variables captured of how isolated people fell during the pandemic and how of a burden they think this pandemic is for them personally.

I believe the paper should be published in PLOS One. The data are original, as there are not that many cross-country panel surveys conducted during the COVID-19 pandemic. The analysis is professionally executed and transparently described in the text. Finally, the paper is making an interesting contribution by looking at the evolution of various political and social attitudes from April to December 2020. As the authors correctly point out in the text, most (if not all) existing papers that look at these attitudes focus on the first wave of the pandemic.

Thank you for the kind words.

That said, I’ve a few comments that the authors should address before publication. They are all quite minor in the sense that they don’t require (much) extra analysis, but they are nonetheless important.

1. As I said, I find the first part of the analysis, in which the authors analyze the evolution of various political and social attitudes during the pandemic, particularly interesting. What is missing though is a measure of whether these changes are statistically significant. Relatedly, Figure 1 could include a confidence interval around each point estimate (not just around those of the pooled variable).

In our revised manuscript, we calculate the significance of time effects and reproduce a version of Figure 1 with confidence intervals around all estimates. We relegate this version to the SI, however, because the only way we could fit in all these intervals without overcrowding the plot was through expanding it’s size (giving each outcome it’s own row).

2. From Figure 1, it also appears that the changes between April and December 2020 concern only handful of variables. At various points in the text (including the title), the authors claim that the “system support” variables decrease over this period. In reality, it’s “only” a few of them, namely satisfaction with the way political institutions/democracy work/s (and also whether people feel like proud citizens, but I’m not sure whether this variable is related to the two others). The authors need to be more precise about what attitudes decrease during the panel survey in the abstract, introduction, and conclusion.

It is true that the previous version of the manuscript has not put sufficient emphasis on the fact that a key system support variable, support for democracy has not decreased. We have added caveats acknowledging this finding in multiple places in the manuscript. Given that we have not predicted this finding, our explanation of it is necessarily speculative, yet, we should note that some comparative politics research has found that a lack of democracy often boosts citizens’ support for it.^[1]^ Accordingly, it is plausible that it is indeed the (unavoidable) democratic deficit of pandemic politics (as reflected in the fall of perceived level of democracy) which boosted support for democracy.

3. At various points in the text, the authors make the point that these “system support” variable had already decreased in April 2020 (i.e., the first wave of their panel survey). To back this claim, they compare the variables in their survey to the same variables in the European and World Values Surveys. This is problematic for at least two reasons. First, the people who responded to the authors’ survey are not the same as those who responded to the values surveys. The variables would then only be comparable if the two surveys were randomly sampling the same population. This is a very unlikely given that the survey modes are different (online vs face-to-face). I know that the authors use entropy matching to address this issue, but it’s only a partial fix given that there might still be some unobserved differences between the two samples. Second, I suppose the values surveys were conducted some time before the start of the pandemic, so it’s not clear whether the observed decrease in the variables of interest is due to the pandemic or any event that happened before it.

For these two reasons, I suggest that the authors remove from the text the argument according to which levels of “system support” already decreased in April 2020. They might say, in the results section, that the data suggest the existence of such decrease, but they do not have the data to make it a solid finding that would then appears in the abstract, introduction and conclusion. We simply do not know whether the trend before April 2020.

We acknowledge that the change between the WVS and our data are less precisely estimated than the trends between our waves. Accordingly, we removed this result from the abstract and conclusion, and added a paragraph to our limitations section explaining why they should be taken with a grain of salt. Yet, we are hesitant to completely remove this finding from the manuscript because – as we state in the limitations section – we believe the parallels in pre-April and post-April changes both in social solidarity (no change) and system support (decline) are notable and make it unlikely that whatever bias is induced by the survey mode completely offsets our main substantive insights.

4. In the second part of the analysis, the authors regress the same social and political attitudes on variables capturing subjective assessments regarding how much a burden the pandemic has been for respondents and how isolated they have felt. They then use two-way fixed effects so that the estimates capture within-respondents and within-waves associations. As I said, the analysis is professionally executed. However, I’m afraid the results cannot be interpreted as “effects”. A key issue is that both the independent and dependent variables are self-reported attitudes. Whereas the two-way fixed effects minimize the possibility that the effect is confounded, it does not address the issue of reverse causality. For example, the authors argue that it’s because respondents fell that the pandemic was a burden for them that their satisfaction with political institutions decreased. But it might be the other way round: Respondents fell that the pandemic was a burden for them because they were unhappy with political institutions. For example, we all know people who were very pessimistic about the impact of the pandemic on their situation (including personal financial situation) although their situation was objectively good. These were also the people who were the most critical of the government.

For this reason, the authors need to be clear in the text that the results of this second part of the analysis are associations and not effects. In some places in the text, they for example use the word “drive”, which is inappropriate. Furthermore, they need to tamper the argument that panel surveys are excellent tools to estimate causal effects especially when one regresses attitudes on attitudes. On p.12, they for example say that that they “constitute a good (perhaps the best) compromise balancing internal validity and generalizability.” This might be true, but that doesn’t mean that they can interpret the regression coefficients as effects. On pp.17-18, they argue that they are “unbiased estimates of causal effects as long as the parallel trends assumption holds”. That’s not correct given that showing the existence of parallel trends does not shut down the possibility of reverse causality.

To be clear: I don’t mean to diminish the value of the paper. I think it’s equally important to show associations between variables. Not every paper needs to have a causal identification strategy. But it’s important to interpret the results for what they are not to misled the reader.

We completely agree with all these insights. We implemented a number of changes to make sure we don’t mislead any of our readers on the strength of internal validity:

1. We revised the problematic sentence about parallel trends, which was always meant to acknowledge the limits in internal validity (see the following paragraph reporting small feedback effects in some (but not all) relationships.
2. We re-read the manuscript and removed words which may give the wrong impression that we claim causally identified effects (“effect”, “drive”, “cause”).
3. We also hope that our numerous caveats on the fact that our results should not be taken as causal estimate (In the methods section, the results section, and the conclusions) will prevent any readers from being misled. Specifically, we state:
   a. "As we are unable to identify or to experimentally induce exogenous shocks in burden and anomie, our causal ambitions must be tempered." p11
   b. "At the same time, these models remain vulnerable to bias from time-varying confounders and reverse causality." p11
   c. "At the same time, our analyses are vulnerable to bias from time-variant confounders or reverse causality" p19
   d. "our estimates regarding the effects of anomie and covid burden are still vulnerable to, for example, time variant confounders ... Future research could manipulate burden and anomie experimentally to estimate unbiased causal effects" p24

5. A key issue with panel surveys is attrition. Many of the respondent who agree to respond to the first Wave might not agree to respond to subsequent waves. The authors do report the attrition rate, but they do not show whether this affects the representativeness of the sample. Was the sample in the latest wave still representative of the population? This is an important question to assess the external validity of the results.

We agree that this is a key issue, which we addressed but did not explain fully. We revised the text to more clearly highlight that Figure OA1 (reproduced below) displays the demographic imbalance between the initial and the final samples. Reassuringly, it displays that although attrition was not random, demographic asymmetries are small and country specific, and they are adjusted by the entropy weights.

Fig OA1 **Proportion of sex, education, age and region in four samples and four countries**. Red marks denote unweighted samples, green marks denote weighted samples. Dots denote the full sample, triangles denote the balanced panel sample (i.e. only those who participated in all 3 waves). The large black cross denotes the population margins.

6. Finally, and this is very minor, but I find that the legend below Figure 3 could be more explicit. Figure 3 has an unusual as the rows show the dependent variables instead of the independent variable. A clear legend would be useful to avoid any confusion.

Thank you for the good suggestion, we have tweaked the legend.

## Reviewer #2

There’s much to like about this manuscript: It studies a relevant topic, uses original and rich data, analyzes this data in a transparent and convincing way and arrives at compelling results. The presentation is straightforward and the entire research process is being made very transparent, with descriptions of the data collection, ethical approval, operationalization, modeling and results, and full replication materials made available on OSF. Plenty of robustness checks substantiate the core results.

Thank you.

My main criticism is that the manuscript seems somewhat under-theorized. While it does provide a (somewhat cursory) review of the literature as well as two psychological mechanisms for explanation, it does not properly conceptualize any of its core independent or dependent variables. What exactly do you mean by “system support”? What by “social solidarity”? How does your measurement capture those concepts?

As far as the literature review is concerned, I think the recent literature on both the relevance of system support for compliance during the pandemic (Bargain and Aminjonov, 2020; Denemark et al., 2022; Weinberg, 2020) and, even more importantly, the effects of the pandemic on system support and societal relations (Anghel and Schulte-Cloos, 2022; Devine et al., 2020; Erhardt et al., 2022; Erhardt et al., 2023; Esaiasson et al., 2021; Kritzinger et al., 2021; Nielsen and Lindvall, 2021; Rump and Zwiener-Collins, 2021; Schraff, 2021) deserves considerably more attention. At the moment, it feels like the authors primarily use the lit review to cite their own papers rather than to discuss the arguments and findings of others.

We agree that the previous version did not adequately cite prior work and we are grateful for the suggestions on recent literature. In our revision, we have expanded the theory section and offer a more comprehensive review of prior work citing each of the important works suggested by the reviewer. Given that much of prior work focuses on rally-around-the-flag effects in the initial weeks or months of the pandemic, we now devote much more attention to these findings in the theory section. This also helped us clarify our contribution – looking at longer effects – and reconcile the results (see point below).

Besides, we also added formal definitions of both “system support” [citizens' approval or disapproval of the political system they live in -- in our case, democracy (p5)] and “social solidarity” (“citizens' beliefs that other citizens' deserve help when in need, and can be generally trusted” [p5]). Besides, in the Methods section, we directly discuss how our measures are operationalizations of these concepts.

A couple of minor points:

- Not sure I agree about using all respondents (even those who dropped out in later waves) for descriptive analysis of outcome variables – what is the rationale for doing so?

Our rationale is to use all available information for the most precise estimates. While our fixed effects models necessarily rely only on respondents who participated in all waves, responses from respondents at a given wave are still informative for calculating the group mean, despite their subsequent dropping out of the sample. That said, reassuringly, the descriptive analyses remain virtually identical if using the balanced panel sample. We added a note on this to the Modeling subsection p. 11.

- How do your results compare to / can be reconciled with the early rally-around-the-flag effects found by others?

This is a good question. While our answer is necessarily speculative, we added the following discussion to the Discussion section stating that we believe the difference is between initial versus long-term effects. We write:

Our findings stand in contrast with prior research demonstrating initial surges to political trust and system support (Bol et al. 2021; Esaiasson et al. 2021), but they are also congruent with findings which show that rally-around-the-flag effects decay fast and depend on the (perceived) performance of the authorities (Erhardt et al. 2022; Kritzinger et al. 2021). pp 21-22

- You mention violations of democratic standards in many countries during the pandemic (p. 3). Could those be an alternative explanation for waning system support?

Yes, this is a good point and indeed, in OA Section D1, we report empirical evidence, which is consistent with this possibility: changes in individuals concern about undemocratic practices as in response to the pandemic correlate with changes in system support variables (primarily satisfaction with the political system and level of democracy). We amended this section with a paragraph acknowledging that “These results are consistent with the possibility that besides the perceived burdens in finance, health, social life and anomie, concerns over democratic rights and freedoms have also contributed to the waning system support throughout the pandemic.”

- In terms of typos, I noticed that “analyses” (as in the plural of “analysis”) is often misspelled as “analyzes”. Also, some special characters in authors’ names aren’t being displayed correctly (e.g., Obradovi´c, Power and Sheehy-Skeffington 2020, p. 4).

Thanks, we corrected these typos.

**References**

Anghel V and Schulte-Cloos J (2022) COVID-19-Related Anxieties Do Not Decrease Support for Liberal Democracy. European Journal of Political Research online first.

Bargain O and Aminjonov U (2020) Trust and Compliance to Public Health Policies in Times of Covid-19.

Denemark D, Harper T and Attwell K (2022) Vaccine Hesitancy and Trust in Government: A Cross-National Analysis. Australian Journal of Political Science 57(2): 145–163.

Devine D, Gaskell J, Jennings W, et al. (2020) Trust and the Coronavirus Pandemic: What are the Consequences of and for Trust? An Early Review of the Literature. Political Studies Review online first.

Erhardt J, Freitag M and Filsinger M (2023) Leaving Democracy? Pandemic Threat, Emotional Accounts and Regime Support in Comparative Perspective. West European Politics 46(3): 477–499.

Erhardt J, Freitag M, Wamsler S, et al. (2022) What Drives Political Support? Evidence from a Survey Experiment at the Onset of the Corona Crisis. Contemporary Politics 28(4): 429–446.

Esaiasson P, Sohlberg J, Ghersetti M, et al. (2021) How the Coronavirus Crisis Affects Citizen Trust in Institutions and in Unknown Others: Evidence from ‘the Swedish Experiment’. European Journal of Political Research 60(3): 748–760.

Kritzinger S, Foucault M, Lachat R, et al. (2021) ‘Rally Round the Flag’: The COVID-19 Crisis and Trust in the National Government. West European Politics 44(5-6): 1205–1231.

Nielsen JH and Lindvall J (2021) Trust in Government in Sweden and Denmark during the COVID-19 Epidemic. West European Politics 44(5-6): 1180–1204.

Rump M and Zwiener-Collins N (2021) What Determines Political Trust during the COVID-19 Crisis? The Role of Sociotropic and Egotropic Crisis Impact. Journal of Elections, Public Opinion & Parties 31(S1): 259–271.

Schraff D (2021) Political Trust during the Covid‐19 Pandemic: Rally around the Flag or Lockdown Effects? European Journal of Political Research 60(4): 1007–1017.

Weinberg J (2020) Can Political Trust Help to Explain Elite Policy Support and Public Behaviour in Times of Crisis? Evidence from the United Kingdom at the Height of the 2020 Coronavirus Pandemic. Political Studies online first.

1. Maseland, Robbert, and André Van Hoorn. "Why Muslims like democracy yet have so little of it." *Public Choice* 147 (2011): 481-496.
